# An Initial Alignment Technology of Shearer Inertial Navigation Positioning Based on a Fruit Fly-Optimized Kalman Filter Algorithm

**DOI:** 10.1155/2020/8876918

**Published:** 2020-10-13

**Authors:** Miao Wan, Zhongbin Wang, Lei Si, Chao Tan, Hao Wang

**Affiliations:** ^1^School of Mechatronic Engineering, China University of Mining & Technology, No. 1 Daxue Road, Xuzhou 221116, China; ^2^CRRC Zhuzhou Locomotive Co., Ltd., Zhuzhou 412001, China; ^3^Jiangsu Key Laboratory of Mine Mechanical and Electrical Equipment, China University of Mining & Technology, No. 1 Daxue Road, Xuzhou 221116, China

## Abstract

The shearer is one of the core equipment of the fully mechanized coal face. The fast and accurate positioning of the shearer is the prerequisite for its memory cutting, intelligent height adjustment, and intelligent speed adjustment. Inertial navigation technology has many advantages such as strong autonomy, good concealment, and high reliability. The accurate positioning of the shearer based on inertial navigation can not only determine its operating position but also measure the direction of movement. However, when inertial navigation is used to locate the shearer in motion, the cumulative errors will occur, resulting in inaccurate positioning of the shearer. The accuracy of the initial alignment is directly related to the working precision of the inertial navigation system. In order to improve the efficiency and accuracy of initial alignment, an improved initial alignment method is proposed in this paper, which uses a fruit fly-optimized Kalman filter algorithm for initial alignment. In order to improve the filtering performance, the fruit fly-optimized Kalman filter algorithm uses an improved fruit fly algorithm to realize the adaptive optimization of system noise variance. Finally, simulation and experiments verify the effectiveness of the fruit fly-optimized Kalman filter algorithm.

## 1. Introduction

Coal is the main energy in China. With the development of the mining industry, coal mine accidents occur frequently, and coal mining technology tends to be unmanned and intelligent. The application of inertial navigation on the shearer has become one of the important mining technologies for unmanned and intelligent coal mining [1, 2], which is used for the rapid and effective autonomous positioning of the shearer. Due to the error accumulation problem in inertial navigation, the initial error should be minimized before the navigation calculation. The accuracy of the initial alignment is directly related to the working precision of the inertial navigation system. Therefore, the rapid and accurate initial alignment becomes a research hotspot [3–5]. For an inertial navigation system, the initial alignment is to determine an initial value such as an attitude matrix at the initial moment. In the initial alignment, using the output information of the inertial component, the appropriate filtering method is selected for initial alignment. Then, the misalignment angle between the calculated navigation coordinate system and the real navigation coordinate system is estimated to correct the attitude matrix, so that the calculated coordinate system and the real coordinate system are obtained as close as possible [6, 7]. Kalman filtering (KF) has a good application for linear system filtering, but Kalman filter (KF) cannot filter nonlinear systems. For the nonlinear systems, the Extended Kalman Filter (EKF) and the Unscented Kalman Filter (UKF) were invented, and their principle is to convert nonlinear systems into linear systems for filtering [8, 9]. The Extended Kalman Filter (EKF) approximates the nonlinear model to a linear model by Taylor expansion while the Unscented Kalman Filter (UKF) approximates the nonlinear model to a linear model through Unscented Transformation (UT); such methods can solve nonlinear model filtering, but they introduce a large amount of computation [10–12].

In recent years, intelligent algorithms have been widely used in the field of artificial intelligence and deep learning, and the more classical ones are genetic algorithms (GA) [13], particle swarm optimization (PSO), and so on [14]. The fruit fly optimization algorithm (FOA) is an intelligent algorithm that seeks global optimization based on fruit fly foraging behavior. The fruit fly itself is superior to other species in sensory perception, especially in the sense of smell and vision, and the olfactory organs can find all kinds of scents floating in the air and even smell food sources 40 kilometers away. Then, after flying near the food location, it can use its sensitive vision to find food and the location where the companions gather and fly in that direction [15, 16]. Compared with other classical intelligent algorithms, the fruit fly optimization algorithm (FOA) has been applied to various fields because of its simple structure, fast convergence speed, and high convergence precision, and it attracted the interest of a large number of researchers. Since the search path of the fruit fly optimization algorithm (FOA) has difficulty in meeting the requirements of practical engineering requirements, many scholars have improved the search path of the fruit fly optimization algorithm (FOA) [17, 18]. The search path of fruit fly optimization algorithm (FOA) is subject to uniform distribution, for the difficulty of meeting the practical engineering application. Literature 19 proposes several improved fruit fly optimization paths and uses improved fruit fly optimization algorithm to optimize the dual-tree complex wavelet algorithm (DTCWT) for denoising in practical engineering applications and achieved reliable results [19]. In the literature 20, Gaussian distribution replaces the original uniform distribution path in fruit fly optimization algorithm (FOA), and it is used to optimize wavelet decomposition (WT) and empirical mode decomposition (EMD) to denoise the cutting sound of the shearer and then improve the recognition accuracy of the shearer cutting state [20]. Since the selection of the system noise variance *Q* parameter of Kalman filter (KF) has a significant influence on the filtering effect [21], the selection of the *Q* is a major difficulty for scholars to study; most of them now use experience to choose *Q*, but, in practical engineering applications, it is difficult to find a suitable empirical value. In this paper, an improved fruit fly optimization algorithm is used to find the appropriate Q value of Kalman filter (KF) and then the improved Kalman filter is used to optimize the initial alignment of the shearer positioning.

The rest of the paper is organized as follows.  Section 2 is the basic theory. In Section 3, the initial alignment method based on the fruit fly-optimized Kalman filter algorithm is presented. The simulation test and physical experiment are described in Section 4, and the conclusions are drawn in Section 5.

## 2. Basic Theory

### 2.1. Kalman Filter

With the development of mathematical theory and technology, many filtering ideas and methods suitable for inertial navigation have emerged, and the classic algorithm is Kalman filter (KF). In the early 1960s, Kalman and Bucy proposed this linear filtering method, which combines the concepts of recursive ideas and state variables. KF can estimate the state quantity of the system according to the observation measurement that can be observed in the system and continuously correct the state value and use the system state equation and observation equation to calculate and recurse the parameters. Since the mean square error obtained by estimating the state quantity is less than or equal to the other estimated mean square error, the KF method is also called the optimal estimation method.

Assume that the state equation and the observation equation of the system are as follows:

(1) $$\begin{array}{c} X\left(k\right)=AX\left(k-1\right)+\Gamma W\left(k-1\right),\\ Z\left(k\right)=HX\left(k\right)+V\left(k\right), \end{array}$$

where *X*(*k*) represents the state vector at system *k*, *A* represents the state transition matrix of the system from *k − *1 to *k*, and *W*(*k − *1) represents the process noise of the system. *Z*(*k*) represents the observation vector at the time of system *k* and *V(k)* represents the observed noise. Γ represents the noise drive matrix of the system and *H* represents the observation matrix. Generally, it is assumed that *W(k − 1)* and *V(k)* are mutually independent white noises, and their covariances are *Q*_*K*_ and *R*_*K*_, respectively, and the correlation covariance of the two is 0. The recursive process of the estimated value $\hat{X}\left(k\left|k\right.\right)$ of the state value *X(k)* at time *k* is as follows.

The predicted equation of the state variables is

(2) $$\begin{array}{c} \hat{X}\left(\left.k\right|k-1\right)=A\hat{X}\left(\left.k-1\right|k-1\right). \end{array}$$

The updated equation for estimating the state variables is

(3) $$\begin{array}{c} \hat{X}\left(\left.k\right|k\right)=\hat{X}\left(\left.k\right|k-1\right)+K\left(k\right)\left[Z\left(k\right)-H\hat{X}\left(\left.k\right|k-1\right)\right]. \end{array}$$

The expression matrix of the filter gain is

(4) $$\begin{array}{c} K\left(k\right)=\frac{P\left(k\left|k-1\right.\right)H^{T}}{\left[HP\left(k\left|k-1\right.\right)H^{T}+R_{k}\right]}. \end{array}$$

The matrix equation for predicting covariance is

(5) $$\begin{array}{c} P\left(k\left|k-1\right.\right)=AP\left(k-1\left|k-1\right.\right)A^{T}+\Gamma Q_{k-1}\Gamma^{T}. \end{array}$$

The updated equation of the covariance matrix is

(6) $$\begin{array}{c} P\left(k\left|k\right.\right)=\left[I-K\left(k\right)H\right]P\left(k\left|k-1\right.\right). \end{array}$$

All the above equations are the recursive process of the Kalman filter. Only *X*0 and *P*0 need to be known, and the optimal estimation value of the state quantity at time *k* can be derived from the observation at the corresponding time.

### 2.2. Fruit Fly Optimization Algorithm

The fruit fly optimization algorithm is a bionic algorithm based on the foraging behavior of fruit fly, and the basic flow of the original fruit fly optimization algorithm can be divided into the following steps:

 
*Step 1*. Initialization algorithm parameters: population size, *sizepop*; maximum number of iterations, *maxgen*; initial position range, LR; and random flight distance, FR; and equation (7) is the initial position calculation of the fruit fly:

(7) $$\begin{array}{c} \left\{\begin{array}{c} X\_\text{axis}=\text{rand}\left(\text{LR}\right),\\ Y\_\text{axis}=\text{rand}\left(\text{LR}\right). \end{array}\right. \end{array}$$

 
*Step 2*. The random direction and distance of the fruit fly searching for food are given, and the random search direction and distance are as shown in the following equation:

(8) $$\begin{array}{c} \left\{\begin{array}{c} X\left(i\right)=X\_\text{axis}+\text{rand}\left(\text{FR}\right),\\ Y\left(i\right)=Y\_\text{axis}+\text{rand}\left(\text{FR}\right). \end{array}\right. \end{array}$$

 
*Step 3*. Since the position of the food cannot be known, the distance from the origin of the coordinate (*Disti*) is estimated in advance, and, then, the reciprocal is used as the taste concentration determination value (*Si*). The calculation formulas of the two are as shown in the following equation:

(9) $$\begin{array}{c} \text{Disti}=\sqrt{X^{2}\left(i\right)+Y^{2}\left(i\right)},\\ S\left(i\right)=\frac{1}{\text{Disti}}. \end{array}$$

*Step 4.* The taste concentration determination value (*S(i)*) is substituted into the taste concentration determination function; the taste concentration (*smell(i)*) of the individual position of the fruit fly is obtained; the minimum concentration value is found; and then they are saved and recorded, as shown in the following equation:
  (10) $$\begin{array}{c} \text{Smell}\left(i\right)=\text{function}\left(S\left(i\right)\right),\left[\text{bestsmell,bestindex}\right]=\min\left(\text{Smell}\left(i\right)\right), \end{array}$$

  where *bestsmell* is the minimum taste concentration, *bestindex* is the corresponding fruit fly individual, and *Smell* is the set of taste concentration in this group.
 
*Step 5*. The latest taste concentration will be compared with the best taste concentration obtained before; if the latest taste concentration is better than the previous generation, the latest fruit fly position and taste concentration will be updated, as in the following equation:

(11) $$\begin{array}{c} \text{smellbest}=\text{bestsemll}\left\{\begin{array}{c} X\_\text{axis}=X\left(\text{bestindex}\right),\\ Y\_\text{axis}=Y\left(\text{bestindex}\right). \end{array}\right. \end{array}$$

  Otherwise, Step 2 to Step 4 will be repeated until a better taste density value is found.
 
*Step 6*. When the taste density value reaches the preset precision value or the run reaches the maximum number of iterations, the loop ends; otherwise, Step 2 to Step 5 will be repeated.

### 2.3. Model of Navigation

**Figure 1:**
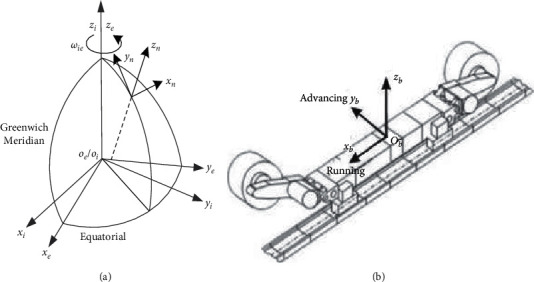
Coordinate systems schematic diagram.

The shearer inertial navigation positioning is to fix the inertial sensor (gyroscope and accelerometer) on the shearer and obtain the angular velocity and acceleration information of the shearer separately after the calculation of the navigation computer, the attitude, speed, and position of the shearer in the navigation coordinate system are obtained. The positional parameters in strap-down inertial navigation system are determined by mutual conversion between different coordinate systems, and several coordinate systems need to be defined, and the required coordinate systems are shown in Figure 1.

The inertial coordinate system (*i*-system) is the measurement reference of the inertial sensor and the origin at the center of the Earth; the coordinate axis has no rotation with respect to the star; the axial direction is defined as *x*_*i*_, *y*_*i*_, and *z*_*i*_; and the *z*_*i*_ is consistent with the direction of the polar axis of the Earth. Earth coordinate system (*e*-system) is fixed to the Earth and the origin also at the center of the Earth. Similarly, each axis is defined as *x*_*e*_, *y*_*e*_, and *z*_*e*_, and the *z*_*e*_ direction is consistent with the direction of the polar axis of the Earth and *x*_*e*_ along the intersection of the Greenwich meridian and the equatorial plane. The *e* system rotates at an angular velocity *ω*_*ie*_ with respect to the *i* system about *z*_*e*_, and *ω*_*ie*_ is the Earth's rotational speed. The navigation coordinate system (*n*-system), also known as the local geographic coordinate system, is located at the position where the navigation system is located, and this paper is set at the end of the working face at the start position of the shearer. The coordinate axes point in the direction of the east (*x*_*n*_), the true north (*y*_*n*_), and the sky (*z*_*n*_) direction (the direction of the local vertical line). The calculated navigation coordinate system (*t*-system) is calculated by calculation, and there is a platform error with the real navigation coordinate system (*n*-system). The carrier coordinate system (*b*-system), which can be called the shearer coordinate system, is fixed with the shearer, and the origin is located at the center of gravity of the shearer. As shown in Figure 1(b), it is an orthogonal coordinate system, and the *z*_*b*_ points are vertically up, the *x*_*b*_ along the direction of advancing, and the *y*_*b*_ points to the direction of running. The angle of rotation about the *x*-axis is called the roll angle, which is recorded as *θ*; the angle of rotation about the *y*-axis is called the pitch angle, which is recorded as *γ*; and the angle of rotation about the *z*-axis is called the yaw angle, which is recorded as *φ*. The attitude angle of the shearer is represented by the transformation relationship between the shearer coordinate system and the navigation coordinate system.

When the shearer is performing inertial navigation, the navigation parameters are obtained through the mathematical relationships, and the calculation errors of mathematical relationships have a close relationship with the system accuracy. Therefore, the correct navigation equations need to be established.

Consider the shearer as a rigid body and set the shearer position vector to *r*. The inertial navigation system has many mechanical arrangement methods and because the shearer runs along the surface of the Earth, this paper selects the arrangement of the navigation coordinate system. The following are the derivation process of the navigation equations based on this arrangement. According to the Coriolis equation, the inertial velocity can be expressed in terms of the Earth's velocity, as shown in the following equation:

(12) $$\begin{array}{c} {\left.\frac{\text{d}r}{\text{d}t}\right|}_{i}={\left.\frac{\text{d}r}{\text{d}t}\right|}_{e}+w_{ie}\times r_{i}. \end{array}$$

*w*_*ie*_ is the angular velocity of the Earth's rotation. Let(d*r*/d*t*)|_*e*_=*v*_*e*_; when equation (12) is derived, in the following equation can be obtained:

(13) $$\begin{array}{c} {\left.\frac{{\text{d}}^{2}r}{\text{d}t^{2}}\right|}_{i}={\left.\frac{\text{d}v_{e}}{\text{d}t}\right|}_{i}+{\left.\frac{\text{d}}{\text{d}t}\left(\omega_{ie}\times r_{i}\right)\right|}_{i}. \end{array}$$

Combining equations (12) and (13) and taking the angular velocity of the Earth's rotation as a constant, the expression can be obtained, as shown in in the following equation:

(14) $$\begin{array}{c} {\left.\frac{{\text{d}}^{2}r}{\text{d}t^{2}}\right|}_{i}={\left.\frac{\text{d}v}{\text{d}t}\right|}_{i}+\omega_{ie}\times v_{e}+\omega_{ie}\times \left[\omega_{ie}\times r\right]. \end{array}$$

It is known that the specific force *f* is the vector sum of the inertial force and the gravitational force, that is, *f*=(d^2^*r*/d*t*^2^)|_*i*_ − *g*. Setting it in equation (14), the following equation can be obtained:

(15) $$\begin{array}{c} {\left.\frac{\text{d}v}{\text{d}t}\right|}_{i}=f-\omega_{ie}\times v_{e}-\omega_{ie}\times \left[\omega_{ie}\times r\right]+g. \end{array}$$

In equation (15), *ω*_*ie*_ × *v*_*e*_ is the Coriolis acceleration and *ω*_*ie*_ × [*ω*_*ie*_ × *r*] is the centripetal acceleration, which cannot be separated from the gravitational acceleration *g* caused by mass gravity. The sum of the acceleration caused by mass gravitation and centripetal force constitutes the local gravity vector; therefore, *g* − *ω*_*ie*_ × [*ω*_*ie*_ × *r*] can be regarded as the local gravity vector, which is represented by *g*_1_. The available expression is as shown in the following equation:

(16) $$\begin{array}{c} {\left.\frac{\text{d}v_{e}}{\text{d}t}\right|}_{i}=f-\omega_{ie}\times v_{e}+g_{1}. \end{array}$$

In the mechanical arrangement of the navigation coordinate system, Earth velocity is expressed as *v*_*e*_^*n*^, and its rate of change relative to the *n*-system can be expressed by the rate of change under the *i*-system, as shown in the following equation:

(17) $$\begin{array}{c} {\left.{\left.\frac{\text{d}v_{e}}{\text{d}t}\right|}_{n}=\frac{\text{d}v_{e}}{\text{d}t}\right|}_{i}-\left[\omega_{ie}+\omega_{en}\right]\times v_{e}. \end{array}$$

In equation (17), *ω*_*ie*_ is the angular velocity of the Earth's rotation and *ω*_*en*_ is the rotational angular velocity of the *n*-system relative to the e-system. Combining equation (16), equation (17) can be expressed as a form in the navigation coordinate system, as shown in the following equation:

(18) $$\begin{array}{c} {\dot{v}}_{e}^{n}=f^{n}-\left[2\omega_{ie}^{n}+\omega_{\text{en}}^{n}\right]\times v_{e}^{n}+g_{1}^{n}. \end{array}$$

Equation (18) is the basic equation of the coal mining machine inertial navigation system. *f*^*n*^ is the specific acceleration in the navigation system, and its component form is $f^{n}={\left[\begin{array}{ccc} f_{E} & f_{N} & f_{U} \end{array}\right]}^{T}$. *v*_*e*_^*n*^ is the projection of the speed of the shearer relative to the Earth in the n-system and the coordinate axes are along the east, north, and local vertical lines. Its component form is $v_{e}^{n}={\left[\begin{array}{ccc} v_{E} & v_{N} & v_{U} \end{array}\right]}^{T}$. *ω*_*ie*_^*n*^ is the angular velocity of the Earth's rotation in the *n*-system, which can be expressed as $\omega_{ie}^{n}={\left[\begin{array}{ccc} 0 & \omega_{ie}\cos\;L & -\omega_{ie}\sin\;L \end{array}\right]}^{T}$, and *L* is the latitude where the *n* system is located. *ω*_*en*_^*n*^ is the projection of the angular velocity of the *n*-system relative to the *e*-system in the *n*-system, which can also be the rotational angular velocity of the *n*-series relative to the *e*-system, and it can be expressed as the rate of change of longitude and latitude: $\omega_{en}^{n}={\left[\begin{array}{ccc} -\dot{L} & \dot{l}\cos\;L & -\dot{l}\sin\;L \end{array}\right]}^{T}$. Since the working environment of the shearer is a fully mechanized face, the deviation between the local gravity vector and the vertical can be ignore, so, *g*_1_ can be expressed as $g_{1}={\left[\begin{array}{ccc} 0 & 0 & g_{1} \end{array}\right]}^{T}$. Combining the above conditions and the algorithm of vector cross multiplication, the scalar representation of equation (19) can be obtained:

(19) $$\begin{array}{c} \left[\begin{array}{c} {\dot{v}}_{E}\\ {\dot{v}}_{N}\\ {\dot{v}}_{U} \end{array}\right]=\left[\begin{array}{c} f_{E}+v_{N}\left(2\omega_{ie}+\dot{l}\right)\sin\;L+v_{U}\left(2\omega_{ie}+\dot{l}\right)\cos\;L\\ f_{N}-v_{E}\left(2\omega_{ie}+\dot{l}\right)\sin\;L+v_{U}\dot{L}\\ f_{U}-v_{E}\left(2\omega_{ie}+\dot{l}\right)\cos\;L-v_{U}\dot{L}+g_{1} \end{array}\right]. \end{array}$$

The rate of change of longitude *l* can be expressed by $\dot{l}=\left(v_{E}/\left[\left(R_{0}+h\right)\cos\;L\right]\right)$ and the rate of change of latitude *L* can be expressed by $\dot{L}=\left(v_{N}/\left(R_{0}+h\right)\right)$. *R*_0_ is the average radius of the Earth and *h* is the altitude at which the navigation coordinate system is located. In addition, there is a relationship: *f*^*n*^=*C*_*b*_^*n*^*f*^*b*^, where *C*_*b*_^*n*^ is the direction cosine matrix, also known as the attitude matrix, which can be used to convert the measured value of the specific force into the navigation coordinate system, and *f*^*b*^ is the measured value of the triaxial accelerometer. Then, equation (20) can also be expressed as

(20) $$\begin{array}{c} \left[\begin{array}{c} {\dot{v}}_{E}\\ {\dot{v}}_{N}\\ {\dot{v}}_{U} \end{array}\right]=C_{b}^{n}f^{b}-\left[\begin{array}{c} 2\omega_{ie}\left(v_{N}\sin\;L+v_{U}\cos\;L\right)+\frac{v_{E}}{\left(R_{0}+h\right)}\left(v_{N}\tan\;L+v_{U}\right)\\ -2\omega_{ie}v_{E}\sin\;L+\frac{v_{U}v_{N}-v_{E}^{2}\tan\;L}{\left(R_{0}+h\right)}\\ -2\omega_{ie}v_{E}\cos\;L-\frac{\left(v_{E}^{2}+v_{N}^{2}\right)}{\left(R_{0}+h\right)}+g_{1} \end{array}\right]. \end{array}$$

## 3. The Proposed Initial Alignment Method

### 3.1. Improved Fruit Fly Optimization Algorithm

Due to the simple structure and fast convergence of the fruit fly optimization algorithm, it is widely used in engineering scheduling, neural network parameter optimization, pattern recognition, signal denoising, and so forth. However, after deep research, domestic and foreign scholars found that the algorithm is easy to fall into local optimum, and its population diversity is less, lacking a mutation mechanism.

The original fruit fly optimization algorithm has two drawbacks:

1. From equation (8), the position of each fruit fly is a random step size at the optimal position of the current population during the generation of the next generation of individual flies. This method only depends on the optimal position of the current population and has nothing to do with its own position, which greatly reduces the diversity of the population and is easy to fall into local optimum.
2. In the process of producing the next generation of fruit fly individuals, the search range of the fruit fly is determined by the random flight distance FR. Choosing a larger random step size FR has a better ability to jump out of the local optimum, but it is easy to cause the search to be too slow. Choosing a smaller FR has a faster convergence rate, but it is easy to fall into the local optimum, so the original FR is difficult to meet the actual requirements.

**Figure 2:**
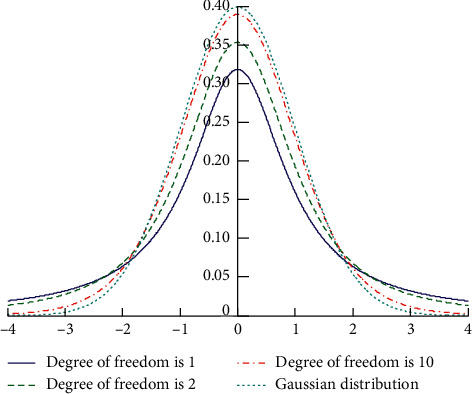
Student's *t*-distribution.

In response to the above shortcomings, two improvements to the fruit fly optimization algorithm are presented in this paper:

1. Fruit flies are divided into two parts. When the next generation group is generated, if the optimal value is not updated for more than two generations, the current optimal position of the fruit fly is accepted, and, otherwise, the current position of the fruit fly itself is accepted.
2. New search path is taken. When the optimal value is not updated for more than two generations, Student's *t*-distribution is used instead of the uniform distribution as the search step. That is, the original *rand* (FR) is replaced by *trnd* (FR) and the number of iterations of the algorithm is used as the degree of freedom of the student's *t*-distribution. On the contrary, according to the characteristic that the frequency is inversely proportional to the wavelength, the frequency variable *f* is introduced and the difference between the current position and the optimal position is multiplied by the frequency variable *f* as the search step. Student's *t*-distribution is shown in Figure 2.

In the first improvement, this improved scheme divides the fruit fly population into two parts, adopting different starting positions, increasing the diversity of fruit flies, and helping the algorithm to jump out of local optimum.

In the second improvement, for path 1, as shown in Figure 2, when the degree of freedom of Student's *t*-distribution is larger, the distribution is closer to the Gaussian distribution. In this paper, the number of iterations is taken as its degree of freedom; as the number of iterations increases, the previous step size of the search path is larger, and the later step size is smaller. And it has a faster search speed and the ability to jump out of local optimum. For path 2, the fruit fly itself moves in the direction of the optimal value by *f* times the linear distance, so the search speed is faster. According to the inverse of the frequency and the wavelength, the frequency variable *f* is used to control the distance of the flight. *f* constantly changes to meet the open flight distance, which has a good effect on the algorithm jumping out of local optimum. The basic process of the improved fruit fly optimization algorithm can be divided into the following steps:

 
*Step 1*. Initialization algorithm parameters: population size, *sizepop*; maximum number of iterations, *maxgen*; initial position range, LR; random flight distance, FR; dimension, *d*; frequency variables, *fmin* and *fmax*; number of iterations, *g*; and so on. Equation (7) is the initial position calculation of the fruit fly and obtains the optimal value of the current population by Step 2–Step 4 in Section 2.2.
 
*Step 2*. When more than two generations do not update the optimal value, the current population optimal position is taken as the starting point, Student's *t*-distribution is used instead of the uniform distribution as the search step, and the degree of freedom is selected as the number of iterations, as shown in the following equation:

(21) $$\begin{array}{c} \left\{\begin{array}{c} X\left(i\right)=X\_\text{axis}+\text{trnd}\left(g,1,d\right),\\ Y\left(i\right)=Y\_\text{axis}+\text{trnd}\left(g,1,d\right). \end{array}\right. \end{array}$$

  Otherwise, the position of the fruit fly itself is taken as the starting point, and the search process is as shown in in the following equation:

(22) $$\begin{array}{c} \left\{\begin{array}{c} f\left(i\right)=f\;\min+{\left(f\;\min-f\;\max\right)}^{*}\text{rand},\\ X\left(i\right)=X\left(i-1\right)+{\left(X\left(i-1\right)-X\_\text{axis}\right)}^{*}f\left(i\right),\\ Y\left(i\right)=Y\left(i-1\right)+{\left(Y\left(i-1\right)-Y\_\text{axis}\right)}^{*}f\left(i\right). \end{array}\right. \end{array}$$

 
*Step 3.* The distance from the origin of the coordinate (Disti) and the taste density judgment value (*S*(*i*)) are estimated, and the calculation process of the two is as shown in equation (9).
 
*Step 4*. The taste concentration determination value (*Si*) is substituted into the taste concentration determination function to obtain the taste concentration (smell(*i*)) of the individual position of the fruit fly; find the minimum concentration value, then, save it, and record its position. The process is as shown in equation (10).
 
*Step 5*. The latest taste concentration will be compared to the best taste concentration obtained before. If the latest taste concentration is better than the previous generation, the latest fruit fly position and taste concentration will be updated as shown in equation (11). Otherwise, Step 2 to Step 4 are repeated until a better taste density value is found.
 
*Step 6.* When the taste density value reaches the preset precision value or reaches the maximum number of iterations, the loop ends; otherwise, Step 2 to Step 5 are repeated.

### 3.2. Initial Alignment Based on a Fruit Fly-Optimized Kalman Filter Algorithm

When using strap-down inertial navigation to locate the shearer, in order to reduce the influence of interference signal and error, it is necessary to study the filtering method, especially the initial alignment. The effective filtering method can not only reduce the operation speed but also improve the system alignment accuracy. Based on the analysis of the advantages and disadvantages of common filtering algorithms, a fruit fly-optimized Kalman filter algorithm is proposed to improve the initial alignment accuracy of the inertial navigation system. In order to improve the filtering performance, the fruit fly-optimized Kalman filter algorithm uses an improved fruit fly algorithm to realize the adaptive optimization of system noise variance, and, then, it is used to conduct initial alignment. The initial alignment is divided into coarse alignment and fine alignment.

#### 3.2.1. Coarse Alignment

In the coarse alignment, the attitude matrix *C*_*b*_^*n*^ is directly estimated by the measurable quantities such as the gravity vector and the Earth angular velocity vector. Given the local longitude *l* and latitude *L*, the components of the gravity acceleration *g* and the Earth rotation speed *ω*_*ie*_ in the navigation system can be determined. Setting a new vector *r*=*g* × *w*_*ie*_, according to the transformation matrix *C*_*b*_^*n*^ between the navigation system and the carrier system, the following expression can be obtained:

(23) $$\begin{array}{c} \left[\begin{array}{c} g^{b}\\ \omega_{ie}^{b}\\ r^{b} \end{array}\right]=C_{n}^{b}\left[\begin{array}{c} g^{n}\\ \omega_{ie}^{n}\\ r^{n} \end{array}\right]. \end{array}$$

There exists

(24) $$\begin{array}{c} C_{b}^{n}={\left(C_{n}^{b}\right)}^{-1}={\left(C_{n}^{b}\right)}^{T}. \end{array}$$

Then, the following equation can be obtained:

(25) $$\begin{array}{c} C_{b}^{n}={\left[\begin{array}{c} {\left(g^{n}\right)}^{T}\\ {\left(\omega_{ie}^{n}\right)}^{T}\\ {\left(g^{n}\times \omega_{ie}^{n}\right)}^{T} \end{array}\right]}^{-1}\left[\begin{array}{c} {\left(g^{b}\right)}^{T}\\ {\left(\omega_{ie}^{b}\right)}^{T}\\ {\left(g^{b}\times \omega_{ie}^{b}\right)}^{T} \end{array}\right]. \end{array}$$

The coarse alignment of *C*_*b*_^*n*^ can be carried out by the abovementioned equation.

#### 3.2.2. Fine Alignment

The coarse alignment results in the transformation matrix between the carrier coordinate system and the calculated navigation coordinate system. Because there are instrument errors in both gyroscopes and accelerometers, the direct calculation results cannot meet the alignment accuracy required by the project. Therefore, after the initial alignment, the precise alignment is required. The fine alignment is based on the coarse alignment, and the platform error angle [*ϕ*×] is accurately estimated; then, obtain the accurate initial attitude matrix *C*_*b*_^*n*^. From the literature [22–24], the error model can be obtained.

The attitude error equation is

(26) $$\begin{array}{c} \dot{\phi }\approx -\omega_{\text{in}}^{n}\times \phi +\delta \omega_{\text{in}}^{n}-\delta \omega_{\text{ib}}^{n}, \end{array}$$

where $\phi ={\left[\begin{array}{ccc} \phi_{E} & \phi_{U} & \phi_{N} \end{array}\right]}^{T}$, *ω*_*in*_^*n*^ is the angular velocity of the *n* series relative to the *i*-system projected under the *n*-system, *δω*_*in*_^*n*^is the calculated error vector of *ω*_*in*_^*n*^, and *δω*_*ib*_^*n*^indicates that the gyro output error in the navigation coordinate system is gyro drift *ε*_*w*_.

The velocity error equation is

(27) $$\begin{array}{c} \delta {\dot{v}}^{n}={\tilde{f}}^{n}-f^{n}+\left(2\omega_{ie}^{n}+\omega_{en}^{n}\right)\times \delta v^{n}-\left(2\delta \omega_{ie}^{n}+\delta \omega_{en}^{n}\right)\times v^{n}-\delta g_{1}^{n}, \end{array}$$

where‘ ∼ 'means the calculated value, the angular velocity of the *n* series relative to the *i* series is projected under the *n* series, *ω*_*ie*_^*n*^ and *ω*_*en*_^*n*^ are obtained in Section 2.3, *δω*_ie_^*n*^ is the calculated error vector of *ω*_*ie*_^*n*^, *δω*_*en*_^*n*^ is the calculated error vector of *ω*_*en*_^*n*^, and *δg*_1_^*n*^ is the calculated error vector of *g*_1_^*n*^:

(28) $$\begin{array}{c} {\tilde{f}}^{n}-f^{n}=C_{n}^{t}f^{n}+\varepsilon_{a}-f^{n}=\left(C_{n}^{t}-I\right)+\varepsilon_{a}, \end{array}$$

where *C*_*n*_^*t*^ means the transformation matrix for the navigation coordinate system and calculated coordinate system, and it can be expressed as

(29) $$\begin{array}{c} C_{n}^{t}=\left(I-\left[\phi \times \right]\right)=\left[\begin{array}{ccc} 1 & \phi_{U} & -\phi_{N}\\ -\phi_{U} & 1 & \phi_{E}\\ \phi_{N} & -\phi_{E} & 1 \end{array}\right]. \end{array}$$

[*ϕ*×] is an antisymmetric matrix of vectors $\left[\begin{array}{ccc} \phi_{E} & \phi_{N} & \phi_{U} \end{array}\right]$; *ϕ*_*E*_, *ϕ*_*N*_, and *ϕ*_*U*_ represent the angle error of east, north, and sky, respectively. *ε*_*a*_ is the zero offset of output error of the accelerometer.

The position error equation is

(30) $$\begin{array}{c} \delta \dot{L}=\frac{\delta v_{N}}{R_{M}+h},\\ \delta \dot{l}=\frac{\delta v_{E}}{R_{N}+h}\sec\;L+\frac{v_{E}}{R_{N}+h}\tan\;L\;\sec\;L\delta L, \end{array}$$

where *R*_*M*_ and *R*_*N*_are the Earth's radii of the meridian circle and the prime vertical circle, *L* and *l* are the longitude and latitude, and *v*_*E*_ and *v*_*N*_ are the east and north velocity with their velocity errors *δv*_*E*_ and *δv*_*N*_, respectively.

Because the initial alignment time is short, the gyro drift and acceleration bias can be considered as random constants. The model of the inertial device can be written as

(31) $$\begin{array}{c} {\dot{\varepsilon }}_{w}=\mathrm{0,}\\ {\dot{\varepsilon }}_{a}=\mathrm{0,} \end{array}$$

where $\varepsilon_{w}=\left[\begin{array}{ccc} \varepsilon_{wE} & \varepsilon_{wN} & \varepsilon_{wU} \end{array}\right]$ and $\varepsilon_{a}=\left[\begin{array}{ccc} \varepsilon_{aE} & \varepsilon_{aN} & \varepsilon_{aU} \end{array}\right]$.

Since the shearer's speed is basically unchanged during actual operation, the system error equation of the inertial navigation of the shearer can be obtained from equation (31):

(32) $$\begin{array}{c} \dot{X}=f\left[X,W\right], \end{array}$$

where the *X* is error vector and *W* is inertial device error vector, and their expressions are as follows:

(33) $$\begin{array}{c} X={\left[\begin{array}{cccccccccc} \delta v_{E} & \delta v_{N} & \phi_{E} & \phi_{N} & \phi_{U} & \varepsilon_{ax} & \varepsilon_{ay} & \varepsilon_{wx} & \varepsilon_{wy} & \varepsilon_{wz} \end{array}\right]}^{T},\\ W={\left[\begin{array}{cccc} \begin{array}{cc} \begin{array}{cc} w_{\delta v_{E}} & w_{\delta v_{N}} \end{array} & w_{\phi_{E}} \end{array} & w_{\phi_{N}} & w_{\phi_{U}} & 0_{5\times 1} \end{array}\right]}^{T}. \end{array}$$

In this paper, two horizontal velocity errors are selected as the observed measurement; they are *δv*_*E*_ and *δv*_*N*_. Then, the observation equation of the system is

(34) $$\begin{array}{c} Z=HX+V. \end{array}$$

In the above equation, $H=\left[\begin{array}{cc} I_{2} & 0_{2\times 8} \end{array}\right]$ and *V* is the system observation noise. In order to conveniently and efficiently use the optimized KF algorithm for recursive calculation, equations (32) and (34) are discretized, and the expression is as shown in the following equation:

(35) $$\begin{array}{c} X_{k}={Ο}_{k,k-1}X_{k-1}+\Gamma_{k-1}W_{k-1},\\ Z_{k}=H_{k}X_{k}+V_{k}. \end{array}$$

In the above equation, *X*_*k*_ is the state vector of the system, *Z*_*k*_ is the observation sequence, *W*_*k*_ is the process noise sequence, *V*_*k*_ is the observed noise sequence, Ο_*k*,*k*−1_ is the state transition matrix, Γ_*k*−1_ is the noise input matrix, and Η_*k*_ is the observation matrix.

The fitness function of the algorithm is selected as

(36) $$\begin{array}{c} \text{fitness}=E\left[\left(x_{P}-x_{t}\right)*{\left(x_{P}-x_{t}\right)}^{T}\right], \end{array}$$

where *x*_*P*_ is the predicted value, *x*_*t*_ is the true value, the fitness is the fitness function, and *E*[*∗*] is the mean value. The basic flow of the improved Kalman filter algorithm can be divided into the following steps:

*Step 1*. Initialization algorithm parameters: population size, sizepop; maximum number of iterations, maxgen; initial position range, LR; random flight distance, FR; dimension, *d*; frequency variables, fmin and fmax; number of iterations, *g*; and so on. Equation (7) is the initial position calculation of the fruit fly and obtains the optimal value of the current population by Step 2 to Step 4 in Section 2.2.
*Step 2.* The optimal value is not updated for more than two generations, and equation (21) is searched by optimization; otherwise, equation (22) is used.
*Step 3*. The distance from the origin of the coordinate (Disti) and the taste concentration determination value (S(*i*)) are estimated, and the calculation process of the two is as shown in equation (9).
*Step 4*. For Kalman filter alignment, the state equation and observation equations are (32) and (34), respectively, and the discretization equation is (35). The taste concentration judgment value is taken as the system noise variance of the Kalman filter algorithm, and the Kalman (S(*i*)) update process is as follows:
  (37) $$\begin{array}{c} \hat{X}\left(k\left|k-1\right.\right)=A\hat{X}\left(k-1\left|k-1\right.\right),\\ \hat{X}\left(k\left|k\right.\right)=\hat{X}\left(k\left|k-1\right.\right)+K\left(k\right)\left[Z\left(k\right)-H\hat{X}\left(k\left|k-1\right.\right)\right],\\ K\left(k\right)=\frac{P\left(k\left|k-1\right.\right)H^{T}}{\left[HP\left(k\left|k-1\right.\right)H^{T}+R_{k}\right]},\\ P\left(k\left|k-1\right.\right)=AP\left(k-1\left|k-1\right.\right)A^{T}+\Gamma S\left(i\right)\Gamma^{T},\\ P\left(k\left|k\right.\right)=\left[I-K\left(k\right)H\right]P\left(k\left|k-1\right.\right), \end{array}$$

and the mean state error and the estimated state variable are calculated as the fitness evaluation standard, and the minimum concentration value is found and saved, and its position is recorded. The process is as shown in the following equation:
  (38) $$\begin{array}{c} \text{smell}\left(i\right)=\text{MSE}\left(\text{Kalman}\left(s\left(i\right)\right)\right),\left[\text{bestsmell,bestindex}\right]=\min\left(\text{Smell}\left(i\right)\right). \end{array}$$

  Among them, bestsmell is the minimum taste concentration, bestindex is the corresponding fruit fly individual, and Smell is the concentration set in this group.
 
*Step 5*. The latest taste concentration will be compared to the best taste concentration obtained before. If the latest taste concentration is better than the previous generation, the latest fruit fly position and taste concentration will be updated as shown in equation (11), and the optimal noise variance will be updated as the following equation:

(39) $$\begin{array}{c} \text{Sbest}=\sqrt{{\left(X\_\text{axis}\right)}^{2}+{\left(Y\_\text{axis}\right)}^{2}},\\ Q=\text{Sbest}. \end{array}$$

  Otherwise, Step 2 to Step 4 are repeated until a better taste density value is found.
 
*Step 6.* When the taste density value reaches the preset precision value or reaches the maximum number of iterations, the loop ends; otherwise, Step 2 to Step 5 are repeated.

**Figure 3:**
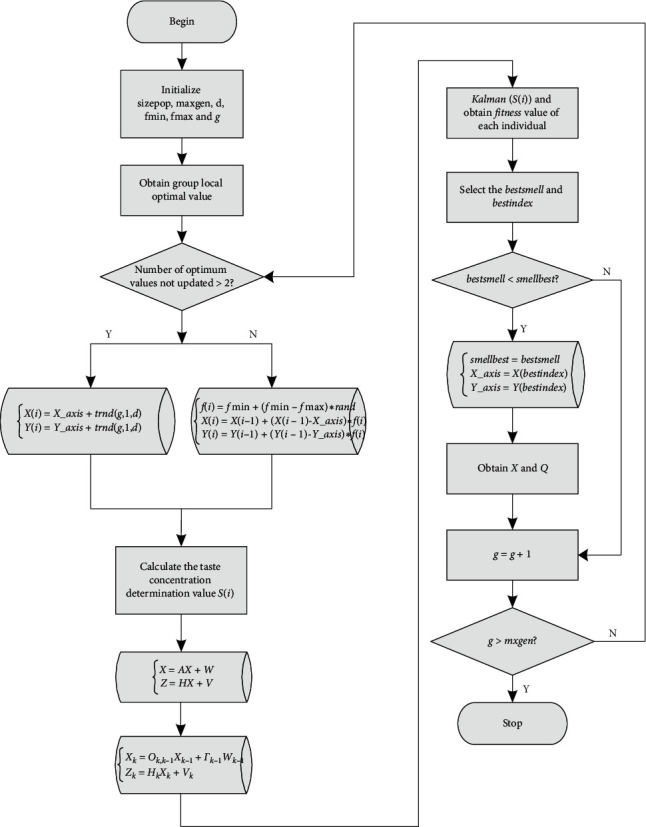
Algorithm flowchart.

The flow chart of the improved algorithm is shown in Figure 3.

The pseudocode for the improved Kalman filter algorithm is shown in Algorithm 1.

## 4. Simulation and Experimental

### 4.1. Simulation

The simulation processing software of this article is MATLAB2014; the hardware configuration is processor Intel Xeon E5506 (2.13 GHz), memory 8G, graphics card NVIDIA Tesla C1060, and hard disk capacity 240G, and software environment is Windows7 (x64).

#### 4.1.1. Simulation of Improved Fruit Fly Optimization Algorithm

This paper selected the original fruit fly optimization algorithm FOA [15] and replaced the original fruit fly step with the Gaussian distribution algorithm GFOA [20], the particle swarm algorithm PSO [14], and the improved fruit fly optimization algorithm IFOA for comparison.

In this simulation, some parameters set for the FOA and GFOA are the same. Some key parameters are given: maximum iteration is 100, population size is 20, and location range is [−1.0, 1.0]. In the IFOA algorithm, the frequency variables fmin and fmax are 0 and 2, respectively, the other parameters are the same as FOA and GFOA.

In the PSO, maximum iteration is 100, population size is 20, two acceleration coefficients are 1.49, inertia weight factor is 0.65, population range is [−5, 5], and velocity range is [−1, 1].

**Figure 4:**
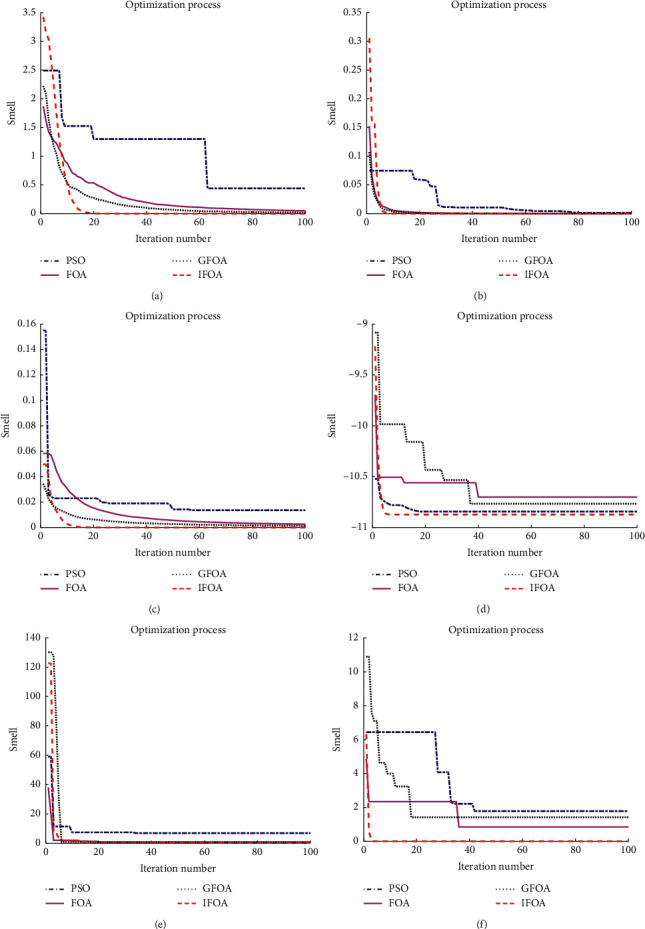
Comparison of the optimization process of the four algorithms. (a) Ackley. (b) Griewank. (c) Zettl. (d) Testtubeholder. (e) Helicalvalley. (f) Wood.

This paper selects the following six test functions to verify the effectiveness of the algorithm. The optimal value of the Ackley function is 0, the optimal value of the Griewank function is 0, the optimal value of the Zettl function is −0.003791, and the optimal value of the Testtubeholder function is −10.8723. The optimal value of the Helicalvalley function is 0, and the optimal value of the Wood function is 0. Comparison of the optimization process of the four algorithms is in Figure 4.

Optimal values of different algorithms are given in Table 1; it can be seen from the above table that the IFOA algorithm proposed in this paper can be the closest to the optimal value of the test function. From Figure 4, the declining curve of the IFOA algorithm proposed in this paper is faster than others, which proves that the IFOA algorithm takes the shortest time to reach the optimal value, and the search process is the fastest.

#### 4.1.2. Simulation of the Initial Alignment Method

To verify the effectiveness of the proposed initial alignment method, this paper selected the original fruit fly optimization algorithm to optimize the Kalman algorithm FOA-KF and replaced the original fruit fly step with the Gaussian distribution to improve the Kalman filter GFOA- KF; particle swarm optimization Kalman filter algorithm PSO-KF is compared with the proposed algorithm IFOA-KF. The parameters set for the FOA-KF, GFOA-KF, IFOA-KF, and PSO-KF are the same as the simulation of improved fruit fly optimization algorithm.

The parameters of the initial alignment are listed in Table 2.

#### 4.1.3. Simulation Results and Analysis

**Figure 5:**
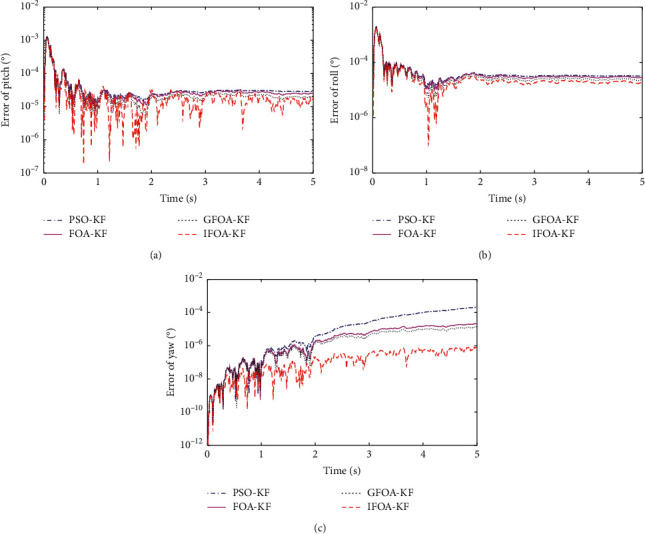
Errors of angles.

Figure 5 is the errors of angles, the best overall is the algorithm IFOA-KF proposed in this article, the best of the remaining three is the GFOA-KF algorithm, followed by the FOA-KF algorithm, and the worst is the PSO-KF algorithm. The errors are shown in Table 3.

As can be seen from Table 3, the algorithm proposed in this paper has the smallest error in the three angles of pitch, roll, and yaw. From the average error point of view, the smallest algorithm is the algorithm proposed in this paper, only 0.000029257. From the analysis of alignment accuracy, it is 45.71% higher than the PSO-KF algorithm, 24.82% higher than the FOA-KF algorithm, and 15.98% higher than the GFOA-KF algorithm. It can be seen that the best algorithm is the algorithm proposed in this paper. The above analysis can conclude that the fruit fly-optimized Kalman filter algorithm is superior to several other algorithms.

There are two reasons for the above phenomenon:

1. The original Kalman filter has a good estimation speed and accuracy for the pitch and the roll angle, but the estimation accuracy and speed of yaw angle are poor, which is determined by the nature of the error. The filtering of *ε*_*wU*_ value is relatively difficult in a short time; this is because it takes the longest time to reflect the velocity observation. When it has a large deviation, the initial error will be continuously amplified after several integrations, resulting in a relatively short estimation speed and accuracy of the yaw angle.
2. The optimized Kalman algorithm uses the intelligent algorithm to adaptively optimize the Kalman *Q* value and judges the appropriate *Q* value based on the variance of the true value and the predicted value. This method improves the filtering accuracy of the Kalman algorithm. The proposed algorithm has made two improvements in the original fruit fly optimization algorithm, which enhances the diversity and the ability to jump out of the local optimum, and improves the search speed.

### 4.2. Experimental

In order to further verify the research results, an initial alignment experiment was conducted in this paper, and the experimental local parameters are shown in Table 4.

**Figure 6:**
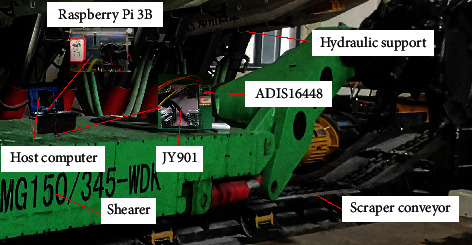
Experimental platform.

The experimental platform is shown in Figure 6.

**Figure 7:**
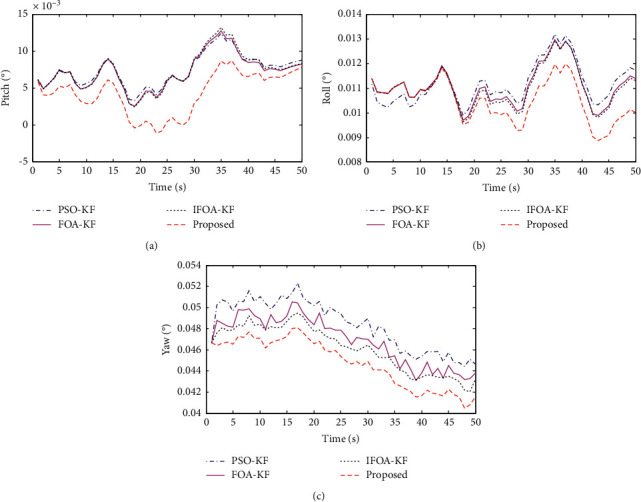
Initial alignment of three angles.

In this experiment, the low-precision inertial measurement module JY901 fixed on the body of the shearer was used to collect the three-axis acceleration and angular velocity of the shearer. In the Raspberry Pi 3b, the initial alignment based on a fruit fly-optimized Kalman filter algorithm was used to process the measured raw data obtained by the JY901. Finally, the result is displayed on the host computer. Use the high-precision inertial measurement module ADIS16448 to obtain the shearer's true attitude angle, the pitch angle is −0.002 degrees, the roll angle is 0.008 degrees, and the yaw angle is 0.004 degrees; they are used as true comparison values to verify the performance of the algorithm in this paper. The initial alignment process at three angles is shown in Figure 7.

The red line in Figure 7 is the initial alignment based on a fruit fly-optimized Kalman filter algorithm proposed in this paper. It can be clearly seen that the algorithm proposed in this paper is superior to several other algorithms in the initial alignment of the yaw angle, pitch angle, and roll angle. The final alignment result of the pitch angle is 0.0078 degrees, the final alignment result of the roll angle is 0.0100 degrees, and the final alignment result of the yaw angle is 0.0415 degrees. The final alignment errors of pitch angle, roll angle, and yaw angle are 0.0098 degrees, 0.0020 degrees, and 0.036 degrees, respectively; the alignment errors in the three directions are all within 0.05 degrees, and the effectiveness of the method proposed in this paper is proved. From the alignment accuracy of three angles, the alignment accuracy of yaw angle is the worst, which is because the initial alignment time of yaw angle is the longest, and the alignment accuracy is poor in a short time. The proposed algorithm in this paper uses the intelligent algorithm to adaptively optimize the Kalman *Q* value that improves the filtering accuracy of the Kalman algorithm. Therefore, it has the best initial alignment accuracy than the other three algorithms.

## 5. Conclusions

The selection of the system noise variance *Q* of the filtering algorithm has a great influence on the filtering effect. Because the fruit fly optimization algorithm has a simple structure and high search efficiency, the fruit fly optimization algorithm was used to optimize the Q value to seek optimal filtering effect in this paper. On the other hand, the fruit fly optimization algorithm also has the disadvantage of being easily trapped in local optimum and so on. Two improvements to the fruit fly optimization algorithm are proposed in this paper. When the fitness function value is not updated for more than two generations, the optimal position is accepted; otherwise, its own position is accepted. Secondly, the original search path is instead by the two new search paths; the diversity and search speed of the fruit fly optimization algorithm are improved and are easier to jump out of local optimum. In the simulation experiment, the good results are achieved by using the improved fruit fly optimization algorithm to optimize the Kalman filter in this paper. Then, the industrial experiment was carried out and the effectiveness of the proposed algorithm is proved.

## Figures and Tables

**Algorithm 1:**
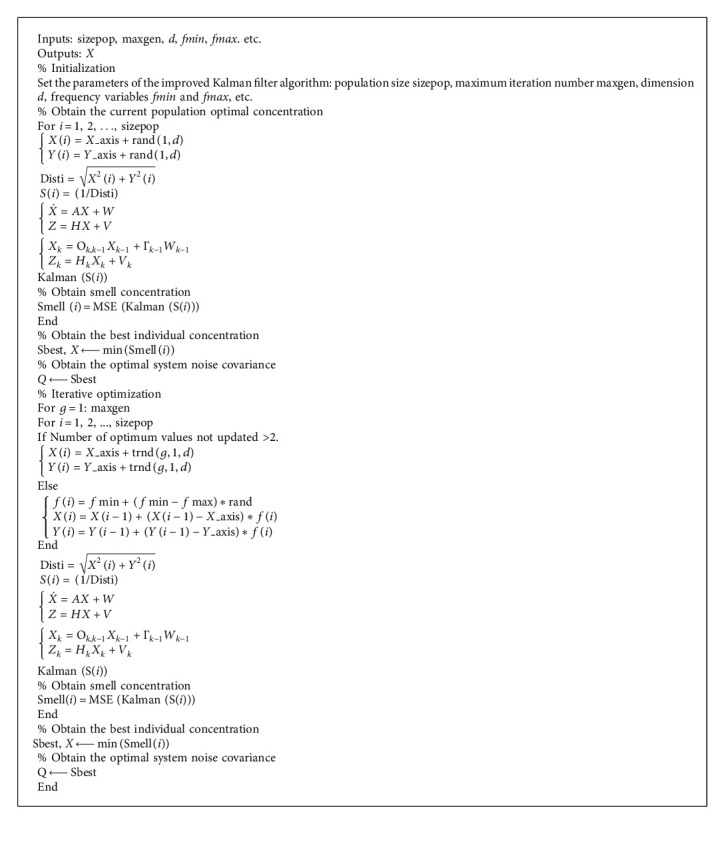


**Table 1:** Optimal value of different algorithms.

Name	PSO	FOA	GFOA	IFOA
Ackley	0.440788	0.049512	0.017546	7.11^∗^10^−15^
Griewank	0.001394	9.29^∗^10^−5^	2.43^∗^10^−5^	3.33^∗^10^−16^
Zettl	0.013575	0.002592	0.001333	3.07^∗^10^−11^
Testtubeholder	−10.843774	−10.700662	−10.766711	−10.872299
Helicalvalley	6.926416	0.739871	0.679324	0.389474
Wood	1.777238	0.852053	1.414687	1.61^∗^10^−4^

**Table 2:** Simulation parameters.

Name	Value	Unit
Longitude	110	deg
Latitude	40	deg
Height	40	m
Average radius	6371393	m
Gravity acceleration	9.7803267714	m/s^2^
Simulation time	5	s

**Table 3:** Comparison of initial alignment errors for the four algorithms.

Name	Pitch	Roll	Yaw	Average error
PSO-KF	0.000050336	0.000065926	0.000045396	0.000053886
FOA-KF	0.000047334	0.000062827	0.000006588	0.000038916
GFOA-KF	0.000042335	0.000057818	0.000004314	0.000034822
IFOA-KF	0.000036726	0.000052809	0.000000242	0.000029257

**Table 4:** Experimental parameters.

Name	Value	Unit
Longitude	117.18	deg
Latitude	39.84	deg
Height	36	m
Average radius	6371393	m
Gravity acceleration	9.7803267714	m/s^2^
Experimental time	50	s

## Data Availability

The data used to support the findings of this study are included within the article.
